# Supplementary material for: Stacking Model‐Based Classifiers for Dealing With Multiple Sets of Noisy Labels
Source: Biom J. 2025 Mar 12;67(2):e70042. doi: 10.1002/bimj.70042 (PMC11898607; doi:10.1002/bimj.70042)
Supplement: Supplementary file 1 — Supporting Information [file BIMJ-67-e70042-s001.zip › density-based-ensemble-model-main/competitors_real_data/netlab-master/netlab-master/help/gpcovarp.htm]

Netlab Reference Manual gpcovarp


# gpcovarp

## Purpose

Calculate the prior covariance for a Gaussian Process.

## Synopsis

```
covp = gpcovarp(net, x1, x2)
[covp, covf] = gpcovarp(net, x1, x2)
```

## Description

`covp = gpcovarp(net, x1, x2)` takes
a Gaussian Process data structure `net` together with
two matrices `x1` and `x2` of input vectors,
and computes the matrix of the prior covariance. This is
the function component of the covariance plus the exponential of the bias
term.

`[covp, covf] = gpcovarp(net, x1, x2)` also returns the function
component of the covariance.

## See Also

`gp`, `gpcovar`, `gpcovarf`, `gperr`, `gpgrad`

---

**Pages:**
Index


---

Copyright (c) Ian T Nabney (1996-9)
